# Case Report: Fruquintinib-induced hyperbilirubinemia: a rare cause of dialyzer filter discoloration in a patient undergoing long-term dialysis

**DOI:** 10.3389/fneph.2025.1586520

**Published:** 2025-07-24

**Authors:** Mercedes Galloway, Alaa S. Awad, Charles W. Heilig

**Affiliations:** Nephrology, University of Florida, Jacksonville, FL, United States

**Keywords:** fruquintinib, dialysis, hyperbilirubinemia, filter discoloration, ESRD, VEGF pathway

## Abstract

We report a case of a 55-year-old male patient with a medical history of cardiorenal syndrome and rectosigmoid colon adenocarcinoma, who started dialysis five years prior to presenting with unusual yellow discoloration of his dialyzer filter during his regular dialysis session. Following a regimen of standard chemotherapy, the patient was initiated on fruquintinib, 5 mg daily for 21 days, as an alternative treatment due to the intolerability of previous agents and failure of malignancy to respond. Shortly after starting fruquintinib, the patient developed hyperbilirubinemia and experienced significant yellow discoloration of the dialysis filter—a phenomenon not previously documented in association with this medication. The absence of dialyzer discoloration during five years of dialysis highlights the temporal relationship between the introduction of fruquintinib and the onset of filter discoloration. [removed some sentence] This case highlights the need for heightened awareness of potential adverse effects of fruquintinib, potentially detectable in patients undergoing dialysis, and aims to contribute to the growing body of literature on the medication’s safety profile.

## Introduction

Managing metastatic colorectal cancer in patients with end-stage renal disease (ESRD) is particularly challenging due to the complexities introduced by renal impairment; hypoalbuminemia and volume overload are common in this population, both of which can alter drug distribution and heighten the toxicity of anticancer agents, making safe and effective dosing especially difficult (1). The multicenter CANDY (CANcer and DialYsis) study highlighted these challenges by showing that among 178 patients with cancer on dialysis, nearly 88% required dose or timing adjustments in their anticancer treatment, with approximately 80% needing administration after dialysis and nearly half requiring overall dose modifications (2).

Most drug safety and pharmacokinetic studies focus on participants with normal kidney function, creating a major knowledge gap for patients with ESRD, who are typically excluded from clinical trials; for example, a review of 94 medication approvals from 2003 to 2007 showed that while just over half included renal impairment data, fewer assessed dialysis patients, and only 41% resulted in dosing guidance based on kidney function—highlighting the need for more consistent, ESRD-specific regulatory standards (3).

Fruquintinib, an oral agent that targets the vascular endothelial growth factor (VEGF) pathway, has shown promise in treating metastatic colorectal cancer, particularly in those who have exhausted standard chemotherapy options and have failed to show improvement (4, 5). Fruquintinib, also known as HMPL-013, is typically used as a third-line therapy for metastatic colorectal cancer; its mechanism specifically involves targeting the tyrosine kinases of 3 different receptors of VEGF (6). While clinical trials such as FRESCO-2 demonstrated that fruquintinib improves overall survival in this population (4), its safety profile in patients with ESRD has not been systematically studied. Existing literature emphasizes common adverse events such as hypertension, hand-foot syndrome, fatigue, and diarrhea (4, 6), though rare or underrecognized adverse effects can emerge in dialysis-dependent individuals. Notably, a prior case report suggested a possible connection between fruquintinib and the development of nephrotic syndrome secondary to thrombotic microangiopathy (TMA), highlighting a rare yet serious renal complication (7). Although the patient in that report did not have ESRD, it emphasizes the vital importance of careful monitoring of blood pressure and proteinuria in all patients treated with anti-VEGF therapies such as fruquintinib, given the potential for significant renal side effects (7). There needs to be cautious monitoring for novel and unexpected adverse effects of fruquintinib in ESRD patients undergoing dialysis, emphasizing the need for further research to better understand its safety and optimize management in this high-risk group.

## Case presentation

A 55-year-old male with a history of cardiorenal syndrome secondary to non-ischemic cardiomyopathy, characterized by an ejection fraction of 40-45% and hypertension, presented to the hemodialysis unit for his scheduled dialysis session. He had been on dialysis for five years with a regimen of three sessions per week (Monday, Wednesday, and Friday), each lasting 3.5 hours, using a left upper extremity arteriovenous fistula. His medical history included colon adenocarcinoma with metastases to the lungs and liver, and unmanaged atrial fibrillation due to thrombocytopenia. During one of his dialysis sessions, the patient started to exhibit yellow discoloration of the dialysis filter and reported abdominal pain. Based on these findings, his session was stopped abruptly and he was advised to seek immediate medical attention at the emergency department.

Upon evaluation in the emergency department, laboratory results revealed hyperbilirubinemia, with a total bilirubin level of 10.3 mg/dL (normal range: 0.1–1.2 mg/dL) and a direct bilirubin of 8.4 mg/dL (normal range: 0.0–0.3 mg/dL). Transaminitis was also noted, with aspartate aminotransferase (AST) at 82 U/L (normal range: 10–40 U/L), Alanine aminotransferase (ALT) at 22 U/L (normal range: 7–56 U/L), and alkaline phosphatase elevated at 152 U/L (normal range: 44–147 U/L). Prior to this episode, his total bilirubin levels had remained within normal limits, with a previous reading of 0.8 mg/dL, despite ongoing treatment for metastatic colorectal cancer. The patient lacked a history of hepatitis, cholelithiasis, biliary obstruction, human immunodeficiency virus, autoimmune or blood disorders that would explain the laboratory abnormalities found.

The background for his colorectal cancer included a diagnosis approximately one year after starting dialysis 5 years ago, following the identification of rectosigmoid colon adenocarcinoma via colonoscopy. Initial treatment consisted of weekly bolus administration of 5-fluorouracil (5-FU) and leucovorin, which continued for over a year but was frequently interrupted due to episodes of acute blood loss anemia. He also underwent partial colectomy, successfully excising 12 lymph nodes, all of which were cancer-free. Post-surgery, he began the FOLFOX chemotherapy regimen (5-FU, leucovorin, and oxaliplatin), but after six months, oxaliplatin was discontinued due to neurotoxic side effects and the patient’s deteriorating clinical condition.

**Figure 1:**
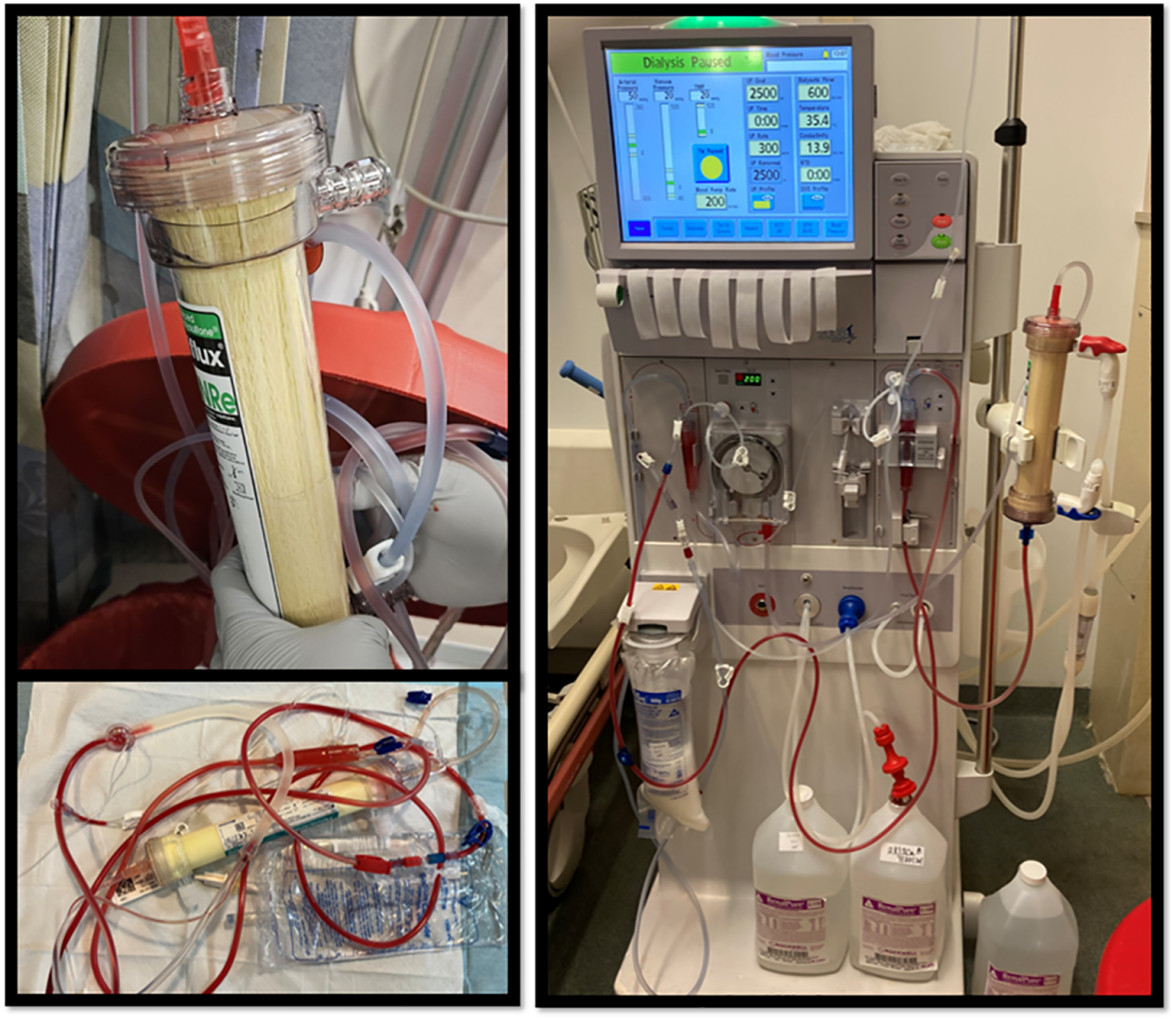
In these images of the dialysis filter, a distinct yellowish hue is observed, which contrasts with the normally white appearance of the filter.

The patient had been receiving treatment for metastatic colorectal cancer for nearly four years, using various chemotherapy agents, and had maintained normal bilirubin levels throughout, without any issues during dialysis sessions so initially it was not clear what had led to his clinical picture. A comprehensive review of medication changes revealed that his oncologist had adjusted his treatment regimen three days prior to a regular dialysis session, where the filter turned yellow (**Figure 1**). The patient had been started on fruquintinib after an outpatient CT scan showed significant disease progression, including notable enlargement of both hepatic and pulmonary metastases. This adjustment was made to provide a more tolerable treatment option, considering the patient’s complex medical history and the ongoing progression of his cancer. He began fruquintinib three days prior to his dialysis session where the filter turned yellow, at a dose of 5 mg daily for 21 days within each 28-day cycle.

Five years prior, the patient’s total bilirubin levels were recorded at 0.7 mg/dL. However, recent lab results after the dialysis filter turned yellow showed a significant increase to 10.3 mg/dL. Notably, the patient had started his new medication a few days before the rise in bilirubin levels was observed. Given his adherence to dialysis and the ability to closely monitor his laboratory values, it was evident that the bilirubin levels began to rise shortly after the initiation of this medication.

After consulting with his oncologist, it was decided to discontinue the medication. Following this adjustment, his total bilirubin levels began to decrease gradually, reaching a value of 5 mg/dL and the dialysis filter lost its yellow color. While the levels did not fully normalize, the trend was considered useful for monitoring purposes, and the elevated bilirubin in the 10 mg/dL range was identified as the likely cause of the yellow discoloration observed in the dialysis filter. He was able to resume dialysis normally without any difficulty. We assessed the urea reduction ratio (URR) and single-pool Kt/V (spKt/V) seven days prior to the initiation of treatment with fruquintinib and again three days following the treatment. Before the initiation of fruquintinib, the URR was 72% and the spKt/V was 1.47. Three days post-treatment, the URR decreased to 67%, reflecting a percentage change of approximately -6.94%, while the spKt/V value slightly reduced to 1.26, showing a percentage change of around -14.29%.

Three weeks after these events, the patient experienced gastrointestinal bleeding and exhibited anemia of chronic disease, which was attributed to his underlying sigmoid adenocarcinoma and end-stage renal disease. During this hospitalization, a computed tomography angiography (CTA) of the abdomen and pelvis was performed, which excluded acute GI bleeding but demonstrated enterocolitis. The patient had been previously admitted for similar symptoms but had left against medical advice, opting to continue dialysis rather than pursue hospice care. There was no recurrent yellow discoloration of the dialysis filter. Upon readmission, the patient reported passing approximately three bags of dark red blood from his colostomy. During this hospitalization, he received one unit of whole blood and two units of packed red blood cells. The patient’s clinical status deteriorated overtime, with development of significant hypotension. This necessitated the temporary cessation of dialysis. Given his declining condition, the patient elected to transition to hospice care for comfort measures and ultimately succumbed to his illness.

## Discussion

The patient in our case had undergone routine dialysis for five years without evidence of dialyzer discoloration until shortly after the initiation of fruquintinib. Notably, the discoloration resolved following the cessation of the drug, accompanied by a concomitant decline in bilirubin levels. This temporal correlation strongly suggests a possible causative association between fruquintinib administration and the onset of both hyperbilirubinemia and dialyzer discoloration. These observations raise the possibility that fruquintinib may interfere with bilirubin metabolism, resulting in its accumulation in patients with ESRD. The close temporal relationship between the initiation of fruquintinib and these clinical findings supports this mechanistic hypothesis.

The patient exhibited no overt physical signs of hyperbilirubinemia—such as jaundice, scleral icterus, or dark urine. Elevations in bilirubin may remain clinically silent and detectable only through routine laboratory monitoring for ESRD patients. A prior case report similarly noted that in anuric ESRD patients, classic manifestations of hyperbilirubinemia are often subtle or absent; for example, tea-colored urine, a common diagnostic clue, may go unnoticed, and jaundice can be obscured by uremic skin discoloration (8). In this population, hyperbilirubinemia is frequently identified solely through periodic lab surveillance rather than physical examination. Although rare and easily overlooked, discoloration of the dialysis filter may serve as an important visual cue of underlying dysfunction, especially in the context of novel medications such as fruquintinib—emphasizing the need for provider vigilance.

Dialyzer discoloration has been documented as a result of multiple factors, including exposure to certain medications (such as rifampin), dyes like fluorescein, and elevated bilirubin levels (8, 9). The appearance of such discoloration should prompt a thorough review of the patient’s medication history, dye exposure, and any underlying conditions that might contribute (8, 9). In the case we present, the exact mechanism underlying the dialyzer discoloration remains uncertain. We hypothesize that unprocessed bilirubin and/or drug–albumin complexes may precipitate onto the hollow fibers of the dialyzer, becoming trapped within the membrane and causing the observed discoloration. However, this proposed mechanism requires further study for confirmation and remains our suggested explanation for the phenomenon observed. Additionally, it is unclear whether the specific composition or type of dialyzer membrane influences this process (10), and further studies are needed to gain more insight.

Beyond aesthetic changes, fruquintinib also appeared to impact dialysis efficiency. We suggest that fruquintinib may impair dialysis performance (whether directly or indirectly with its interactions with the dialysis filter) and therefore warrants close monitoring in patients with ESRD, particularly those with borderline clearance parameters at baseline. Baseline assessments of the patient’s dialysis efficiency over the past five years demonstrated adequate clearance, with a urea reduction ratio (URR) of 72% and a single-pool Kt/V (spKt/V) of 1.47. However, three days following initiation of fruquintinib, URR decreased to 67% (a 6.94% reduction) and spKt/V declined to 1.26 (a 14.29% reduction), indicating a measurable decrease in dialysis efficacy. These modest yet notable changes occurred despite consistent dialysis session duration and patient compliance, minimizing confounding variability. The simultaneous decline in both URR and spKt/V suggests a potential pharmacologic effect of fruquintinib on solute clearance, possible interference with dialysis membrane function or perfusion dynamics. Although the clinical significance of this observation remains to be fully elucidated, it warrants cautious attention.

A potential explanation for the observed reduction in dialysis efficiency is the accumulation of bilirubin within the dialysis membrane. Bilirubin’s poor water solubility and strong protein binding limit its removal by conventional dialysis. High-flux dialyzers, designed to allow small solutes to pass while restricting larger molecules such as albumin, may become overwhelmed by elevated levels of protein-bound bilirubin or fruquintinib-protein complexes. These complexes can precipitate or become trapped within the hollow fibers, impairing membrane function and reducing solute clearance. Fruquintinib itself, likely highly protein-bound, may contribute further to this burden, compounding the decline in dialysis efficacy. Albumin dialysis—an extracorporeal liver support therapy exemplified by systems such as the Molecular Adsorbent Recirculating System (MARS) and Single-Pass Albumin Dialysis (SPAD)—was developed to address the limitations of traditional dialysis, which primarily removes water-soluble toxins, by using albumin as both the dialysis medium and a carrier molecule (11). These therapies enhance clearance of protein-bound and water-insoluble toxins through albumin-rich dialysate and adsorption columns containing charcoal and anion-exchange resins (11–13). This key distinction explains the unexpected hyperbilirubinemia and dialyzer discoloration observed in this case, as standard dialysis membranes struggle to manage the complex interplay between elevated bilirubin and drug-albumin complexes, leading to membrane overload. While albumin dialysis may offer therapeutic advantages when conventional dialysis is insufficient, particularly for protein-bound toxins such as bilirubin and fruquintinib, the effects of albumin dialysis on the drug’s metabolism and clearance remain uncertain, and practical application of these modes of dialysis is often limited by availability and practicality.

Based on our findings, we recommend that clinicians administering fruquintinib to patients undergoing dialysis implement enhanced monitoring protocols. Specifically, regular assessments of liver function tests and total bilirubin—are essential, as fruquintinib has been associated with hepatotoxicity and hyperbilirubinemia (14, 15). While SPAD and MARS are designed to remove protein-bound toxins like bilirubin, their impact on fruquintinib clearance remains uncertain. If fruquintinib is also highly protein-bound, these systems might inadvertently reduce its efficacy by enhancing its clearance. Therefore, we cannot currently recommend their use for this purpose. Furthermore, given the limited data on fruquintinib use in ESRD, we advocate for additional research to evaluate its safety and pharmacokinetics within this population. Studies should focus on determining appropriate dosing adjustments based on GFR and assessing the drug’s clearance during various dialysis modalities. Until such data are available, clinicians should exercise caution, ensuring cautious monitoring of hepatic function and being prepared to modify treatment strategies in response to adverse effects. We hope that future clinical trials will provide more comprehensive guidelines to optimize the safe use of fruquintinib in dialysis-dependent patients.

## Conclusion

Fruquintinib, a novel oral therapy targeting the VEGF pathway for metastatic colorectal cancer, has been largely studied in patients with normal kidney function, leaving its effects on those with ESRD poorly understood. This case reveals a rare instance of fruquintinib-induced hyperbilirubinemia, marked by yellow discoloration of the dialysis filter and a modest decline in renal clearance efficiency, as seen in changes to urea reduction ratio and spKt/V. The temporal link between the drug’s initiation, the development of hyperbilirubinemia, its resolution after discontinuation, and impaired dialyzer urea clearance highlights fruquintinib’s impact on bilirubin metabolism and dialysis function. These findings stress the importance of further research into its safety in ESRD and close monitoring of patients for complications such as bilirubin abnormalities and dialyzer discoloration to ensure safe and effective care.

## Data Availability

The datasets presented in this study can be found in online repositories. The names of the repository/repositories and accession number(s) can be found below: https://www.researchgate.net/publication/385746559_Title_Fruquintinib-Induced_Hyperbilirubinemia_A_Rare_Cause_of_Dialyzer_Filter_Discoloration_in_a_Patient_Undergoing_Long-Term_Dialysis.
